# Polydopamine-assisted versatile modification of a nucleic acid probe for intracellular microRNA imaging and enhanced photothermal therapy†

**DOI:** 10.1039/c8ra00261d

**Published:** 2018-02-12

**Authors:** Aixian Zheng, Xiaolong Zhang, Yanbing Huang, Zhixiong Cai, Xiaolong Liu, Jingfeng Liu

**Affiliations:** The United Innovation of Mengchao Hepatobiliary Technology Key Laboratory of Fujian Province, Mengchao Hepatobiliary Hospital of Fujian Medical University Fuzhou 350025 P. R. China drjingfeng@126.com; The Liver Center of Fujian Province, Fujian Medical University Fuzhou 350025 P. R. China; Liver Disease Center, The First Affiliated Hospital of Fujian Medical University Fuzhou 350005 P. R. China

## Abstract

MicroRNAs play an important role in various biological processes, and their aberrant expression is closely associated with various human diseases, especially cancer. Real-time monitoring of microRNAs in living cells may help us to understand their role in cellular processes, which can further provide a basis for diagnosis and treatment. In this study, polydopamine was used to assist the versatile modification of a nucleic acid probe for intracellular microRNA imaging and enhanced photothermal therapy. Polydopamine can be covalently linked with a thiol-terminated nucleic acid probe through the Michael addition reaction under slightly alkaline conditions. This modification is mild and can be performed directly in an aqueous solution, which can better resist hydrolysis than the traditional modification processes, resulting in a nanoprobe with better stability and higher loading of nucleic acids. This prepared nanoprobe can easily enter cells without transfection agents and then realize the imaging of intracellular miRNA through fluorescence restoration. Moreover, the coating of PDA can enhance the photothermal conversion efficiency of the nanoprobe, making it suitable for photothermal therapy of cancer. It is expected that the PDA-based versatile modification can help to construct a promising platform for tumor imaging and treatment.

## Abstract 2

Polydopamine can assist the versatile modification of a nucleic acid probe for intracellular miRNA responsed fluorescence imaging and enhanced photothermal therapy.

## Graphical abstract

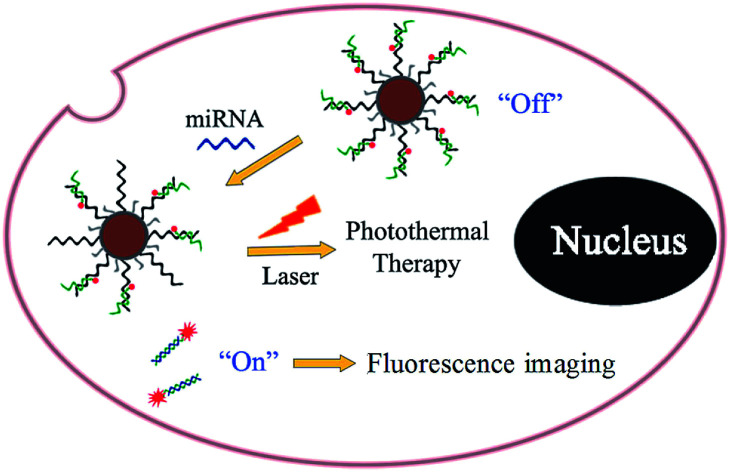

## Introduction

MicroRNAs (miRNAs) are a class of endogenous noncoding RNAs with about 18–24 nucleotides, which can post-transcriptionally regulate gene expression and play important roles in various biological processes such as cell proliferation, migration, differentiation, and apoptosis.^1^ Growing evidence indicates that the aberrant expression of miRNA is closely associated with some major human diseases, especially cancer. This makes miRNA a potential biomarker for diagnostic and prognostic applications. miRNA can also provide new ideas, such as gene silencing and the use of inhibitors, for cancer treatment through the regulation of its expression.^2^ Currently, a number of methods, including real time polymerase chain reactions, northern blot method, and DNA microarrays, have been widely applied for miRNA analysis.^3^ However, these methods are not suitable for monitoring the expression of intracellular miRNA in living cells. Due to the rapid development of optical imaging techniques, several imaging methods^4^ have been established to visualize intracellular miRNA, which may help us to understand the role of intracellular miRNA in cellular processes and thus further provide a basis for diagnosis and treatment of cancer.

Over the past decade, nanomaterials have been widely used in biosensing, drug delivery, cancer diagnosis and treatment.^5^ Moreover, nanomaterial-based nucleic acid nanoprobes, such as magnetic nanomaterials,^6^ silicon nanomaterials,^7^ gold nanomaterials,^8^ and MnO_2_ nanosheets,^9^ have attracted significant attention for intracellular RNA imaging. Among these, gold nanoparticles (AuNPs) are most commonly used. There are two main types of AuNP-based nucleic acid nanoprobes: nanobeacons^10^ and nanoflares.^11^ These nanoprobes possess many interesting properties, including the ability to enter cells without transfection agents and resistance towards enzymatic degradation. The modification of nucleic acid nanoprobe onto the surface of nanomaterials are mainly based on non-specific adsorption or active groups, such as *N*-hydroxysuccinimide (NHS), or the formation of the Au–S bond. This modification may easily generate false positive signals due to protein binding, reduced glutathione (GSH) competition, and other processes.^12^ Moreover, the active group-based modification is easily subjected to hydrolysis, leading to complexity and inefficiency in the modification.

Dopamine, an important neurotransmitter, is also a small molecule mimetic of adherent proteins secreted by mussels. It has been reported that dopamine can self-polymerize to form polydopamine (PDA) under mildly alkaline conditions that can spontaneously deposit on virtually any surface.^13^ Furthermore, the catechol and amino functional groups on the PDA surface enable conjugation of biomolecules. Under slightly alkaline conditions, the chemical equilibrium between catechols and quinones will shift towards the latter such that they can be linked with nucleophiles, such as amine and thiol groups, through Schiff base or Michael addition reactions.^14^ It has been reported that PDA can be covalently linked with DNA containing nucleophilic groups, such as thiol and amine groups, to prepare DNA microarrays.^15^ This PDA-based modification is mild and can be performed directly in an aqueous solution, which can better resist hydrolysis than the modification based on active groups, such as NHS and maleimide, and thus avoid the complexity and inefficiency of the traditional modification processes.

Moreover, PDA possesses good biocompatibility, which makes it suitable for biomedical applications. PDA can act as a fluorescence quencher for fluorophores close to its surface. It has been reported that PDA can adsorb nucleic acid probes to develop imaging methods for intracellular mRNA, ATP, and other biological molecules.^16^ Because of its strong near-infrared absorbance and high photothermal conversion efficiency, PDA can also be used as a photothermal reagent for cancer treatment.^17^ It is expected that these features of PDA can be used to construct a promising platform for tumor imaging and treatment.

**Scheme 1:**
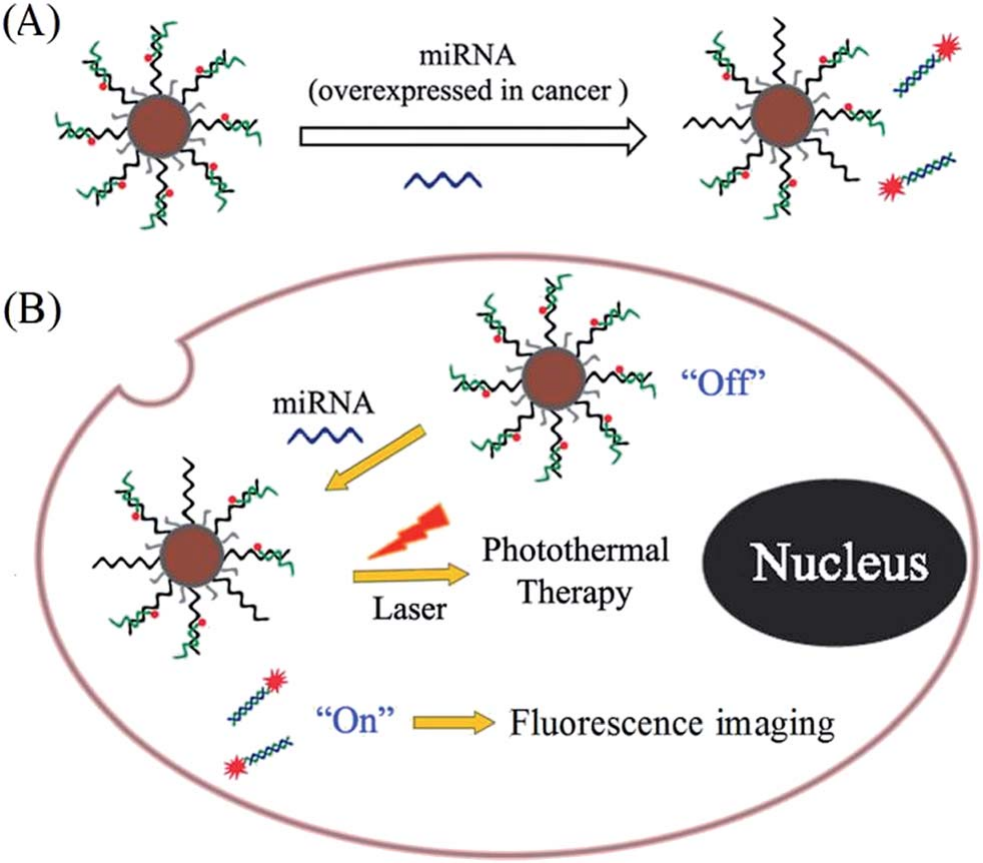
Schematic of polydopamine-assisted versatile modification of a nucleic acid probe for miRNA response-based fluorescence imaging and enhanced photothermal therapy.

In this study, PDA was used to assist the versatile modification of nucleic acid probes for miRNA response-based fluorescence imaging and enhanced photothermal therapy (Scheme 1). As a proof-of-concept, AuNPs are used as a supporter, and miR-21 is chosen as a target, which is one of the most important endogenous miRNA involved in the biological progress of human cancer. Under weak alkaline conditions (pH 8.5), dopamine can self-polymerize on the surface of AuNPs to form PDA-functionalized AuNPs (AuNPs@PDA), which can then be covalently linked with thiol-terminated DNA through the Michael addition reaction. The thiol-terminated DNA is pre-hybridized with FITC-labelled antisense probe; thus, the fluorophores are in close proximity to the surface of AuNPs@PDA, resulting in fluorescence quenching. To increase the loading efficiency, NaCl solution is added during the modification process to increase the electrostatic repulsion between nucleic acid probes. In this way, the prepared nanoprobe (AuNPs@PDA-dsDNA) can exhibit good stability and a high loading of nucleic acid probes. The strand displacement reaction is usually used for nucleic acid-based biosensing and imaging. This reaction is commonly initiated by an overhanging single-stranded DNA region (toehold), which can facilitate branch migration and displace one of the DNA strands from the duplex through a thermodynamically driven process.^18^ Upon the addition of target miRNA to the prepared nanoprobe, the unhybridized region of the FITC-labelled antisense probe can serve as a toehold to hybridize with the target; this leads to the release of their hybrids through a branch migration process and thus fluorescence restoration. This prepared nanoprobe can easily enter cells without transfection agents and thus realize the imaging of intracellular miRNA through fluorescence restoration. It is worth mentioning that the PDA coating can also enhance the photothermal conversion efficiency of the nanoprobe, making it suitable for photothermal therapy of the cancer. This type of nucleic acid nanoprobe is also suitable for other miRNA response-based imaging and treatment by simply changing the relevant nucleic acid sequence.

## Experimental

### Materials and apparatus

Chloroauric acid (HAuCl_4_) and dopamine hydrochloride were purchased from Sigma-Aldrich Chemical Co. Tris(2-carboxyethyl)phosphine hydrochloride (TCEP) was purchased from Aladdin. 4′,6-Diamidino-2-phenylindole dihydrochloride (DAPI) was purchased from Dojindo Molecular Technologies. The cell counting kit-8 (CCK-8) was purchased from Dojindo Laboratories. A live–dead viability/cytotoxicity kit (Calcein AM/EthD-1) was purchased from life technologies, Inc. Nucleic acids used in this study were synthesized by Shanghai Sangon Biotechnology Co. Their sequences were thiol-terminated DNA: 5′-SH-AAAAAAAAAACCCTATAGCTTATCAGAC-3′; FITC-labelled antisense probe: 5′-TCAACATCAGTCTGATAAGCTATAGGG-FITC-3′; miRNA-21 target: 5′-TAGCTTATCAGACTGATGTTGA-3′; miRNA-21 target with single-base mismatch: 5′-TAGCTTATCAGTCTGATGTTGA-3′; miRNA-21 target with three-base mismatch: 5′-TAGCTTTTCAGTCTGAAGTTGA-3′, irrelevant sequence: 5′-GCTAGAGATTTTCCACACTGACT-3′; thiol-terminated short DNA: 5′-SH-AAAAA-3′; and miR-21: 5′-UAGCUUAUCAGACUGAUGUUGA-3′. All other reagents were commercially available and used as received. Ultrapure water was used in all the runs, which was obtained from a Millipore water purification system (Milli-Q).

The absorbance signals were measured by a microplate reader (Spectra Max M5, Molecular Devices). The fluorescence signals were measured by a fluorescence spectrophotometer (Agilent Technologies, Malaysia). The particle size estimated by dynamic light scattering (DLS) was measured by Zetasizer Nano ZS (Malvern Instruments, UK). The temperature of all solutions were measured by a thermocouple microprobe (STPC-510P, Xiamen Baidewo Technology Co., China). Laser irradiation at 670 nm was performed by a continuous-wave diode laser (Beijing Kaipulin Optoelectronic Technology Co., China). The fluorescence imaging was performed by a confocal laser scanning fluorescence microscope (Zeiss LSM780).

### Cell culture

The human cervical cancer cell line (HeLa) and the human hepatocellular carcinoma cell line (HepG2) were maintained as a monolayer culture in an RPMI-1640 medium. The HepG2 cells were obtained from Shanghai Cell Bank of the Chinese Academy of Sciences. The human normal liver cells (LO2) were maintained as a monolayer culture in the DMEM. They both contained 10% fetal bovine serum (FBS) (ExCell Bio, China) and 1% penicillin–streptomycin (Gibco BRL, USA).

### The preparation of AuNPs

AuNPs, about 13 nm in diameter, were prepared using the previously reported methods with some modifications.^19^ In brief, 50 mL of 1 mM HAuCl_4_ was heated to reflux at 130 °C. Then, 5 mL of 38.8 mM trisodium citrate was added quickly under stirring, and the solution changed from yellow to deep red. The mixture was kept refluxing and stirring for an extra 20 min and then slowly cooled down to room temperature. The obtained AuNP solution was stored at 4 °C. The size and morphology of AuNPs were verified by transmission electron microscopy (TEM) and DLS. The AuNP concentration has been estimated by absorbance spectroscopy, which has an extinction coefficient of 2.7 × 10^8^ M^−1^ cm^−1^ at 520 nm.

### Polydopamine-assisted modification of the nucleic acid probe

The AuNP solution was concentrated by centrifugation and redispersed in 10 mM Tris–HCl buffer (pH 8.5). Then, about 16 nM AuNPs were mixed with 0.05 mg mL^−1^ dopamine hydrochloride and stirred for 4 h. The polydopamine-functionalized AuNPs (AuNPs@PDA) were obtained by centrifugation at 14 000 rpm for 20 min and then washed three times with Tris–HCl buffer. Modification of the nucleic acid probe onto AuNPs@PDA was conducted as follows. Briefly, 24 μL of 100 μM thiol-terminated DNA was activated by an equal amount of TCEP first and then mixed with 28.8 μL of 100 μM FITC-labelled antisense probe (1 : 1.2) in PBS buffer (137 mM NaCl, 10 mM phosphate, 2.7 mM KCl, pH 7.4). The solution was maintained at 90 °C for 5 min and then slowly cooled down to room temperature. The solution was stored for several hours to allow sufficient hybridization. The formed DNA duplexes were added to 400 μL of 16 nM AuNPs@PDA and shaken gently. The solution was adjusted with NaCl solution to a final concentration of 0.15 M. After further incubation overnight, 24 μL of 100 μM thiol-terminated short DNA was added to the solution for 2 h to block the surface. After this, the nanoprobe solution was centrifuged at 14 000 rpm for 20 min and then washed three times with PBS. The nanoprobes were resuspended in PBS and stored at 4 °C before use.

The loading amount of nucleic acid probes on the nanoparticle surface was determined from the fluorescence of the labeled FITC. The standard curve was obtained using known concentrations of nucleic acid probe, with the fluorescence intensity as the ordinate and the concentration of nucleic acid probe as the abscissa. After modification, the fluorescence signal of the supernatant was converted to the concentration of the corresponding nucleic acid probe using the standard curve. After calculation, the number of nucleic acid probes per AuNPs@PDA can be obtained.

### The nanoprobe for *in vitro* fluorescence response to the miR-21 target

For detection of the miR-21 target, the nanoprobe was diluted to 1 nM in PBS buffer (137 mM NaCl, 10 mM phosphate, 2.7 mM KCl, pH 7.4) and incubated with various concentrations of miR-21 target for 1 h. After reaction, the fluorescence emission spectra of each solution were obtained with excitation at 480 nm and emission from 500 nm to 650 nm. The final concentrations of the miR-21 target varied from 5 nM to 100 nM. In the control experiments, the miRNA-21 target with single-base mismatch, miRNA-21 target with three-base mismatch, and irrelevant sequence were used instead of the miRNA-21 target under the same conditions for the detection of the miRNA-21 target.

### The nanoprobe for miRNA response-based fluorescence imaging

HeLa cells were seeded in a 35 mm glass-bottom Petri dish and then incubated under a humid atmosphere (5% CO_2_) at 37 °C for 24 h. After washing three times with PBS, they were incubated with either 1 nM AuNPs@PDA-dsDNA or the nanoprobe containing mismatched antisense probe for 2 h to allow cellular uptake. Subsequently, they were washed three times with PBS and then fixed with 4% paraformaldehyde for 10 min. The nuclei of the cells were stained with 2.0 μM DAPI. Finally, the stained cells were imaged by a confocal laser scanning fluorescence microscope with a 488 nm laser excitation for FITC-labelled antisense probe and a 405 nm laser excitation for DAPI.

### Photothermal effect induced by 670 nm laser irradiation

The photothermal conversion capabilities of the prepared AuNPs and AuNPs@PDA induced by 670 nm laser irradiation were evaluated by measuring the temperature changes of their solution at different concentrations (0, 1, 2, 4, and 8 nM). Briefly, 1.0 mL of each solution was irradiated by a 670 nm laser (1 W cm^−2^) for 5 min, and the temperature change of each solution was measured by a thermocouple microprobe. Moreover, the photothermal conversion capability of water was measured for comparison.

### Cytotoxicity and photothermal therapy effect

The cytotoxicity of the prepared nanoprobes was evaluated on LO2 cells, HepG2 cells, and HeLa cells using CCK-8. The corresponding cells were seeded in 96-well plates and then incubated under a humid atmosphere (5% CO_2_) at 37 °C for 24 h. After washing three times with PBS, they were further incubated with a fresh culture medium containing different concentrations (0, 1, 2, 4, 6, and 8 nM) of AuNPs@PDA-dsDNA for 24 h. After washing with PBS to remove non-internalized nanoprobes, 90 μL of the culture medium and 10 μL of the CCK-8 solution were added and incubated for 1 h at 37 °C. The absorbance at 450 nm of each well was measured to calculate the corresponding cell survival rates.

The photothermal ablation ability of AuNPs@PDA-dsDNA against HeLa cells was also measured using CCK-8, and AuNP-dsDNA was used for comparison. Typically, HeLa cells were seeded in a 96-well plate and then incubated under a humid atmosphere (5% CO_2_) at 37 °C for 24 h. After washing three times with PBS, they were further incubated with a fresh culture medium containing different concentrations (0, 1, 2, 4, 6, and 8 nM) of AuNP-dsDNA and AuNPs@PDA-dsDNA for 2 h. After being washed with PBS to remove non-internalized nanoprobes, the cells were irradiated with a 670 nm laser (1 W cm^−2^) for 5 min. Then, the corresponding cell survival rates were measured using CCK-8. To further evaluate the photothermal therapy ability of AuNP-dsDNA and AuNPs@PDA-dsDNA, the live–dead viability/cytotoxicity kit was used to stain the cells after laser irradiation. The corresponding cells were stained with 4.0 μM EthD-1 and 2.0 μM calcein AM for the visualization of dead and live cells.

## Results and discussion

### Characterization of the prepared nanoprobe (AuNPs@PDA-dsDNA)

**Fig. 1:**
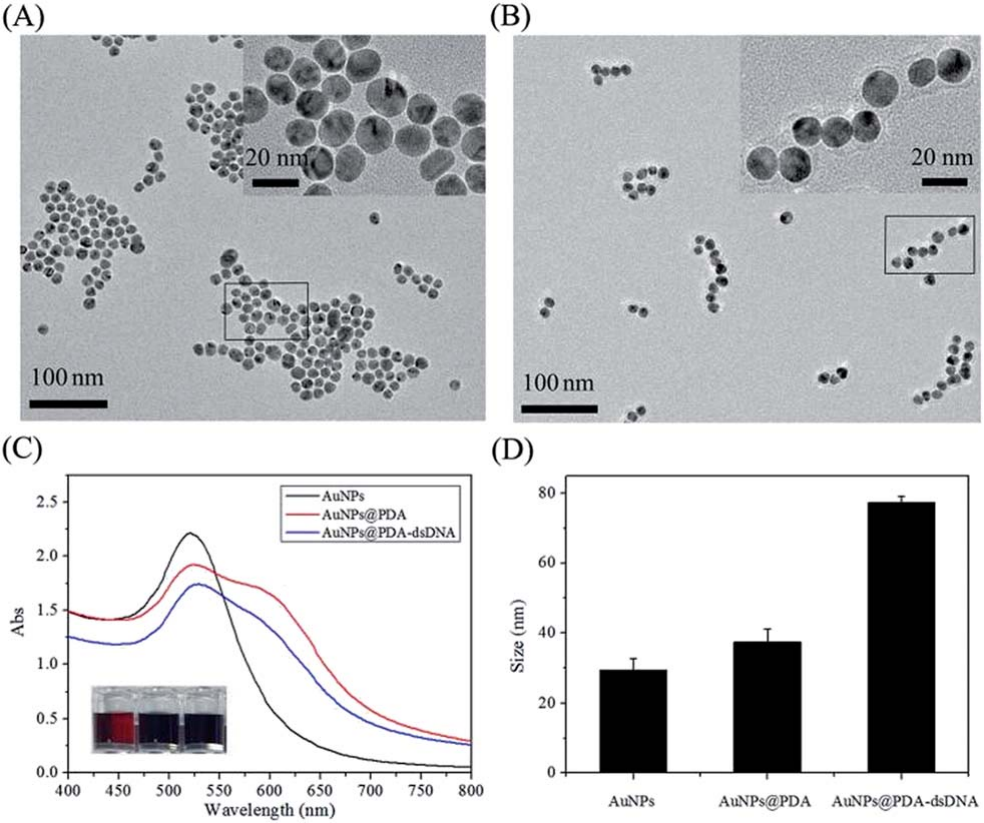
TEM image of AuNPs (A) and AuNPs@PDA (B); (C) the absorbance spectra of AuNPs, AuNPs@PDA, and AuNPs@PDA-dsDNA, and the inset image showing the corresponding colorimetric change; (D) hydrodynamic size of AuNPs, AuNPs@PDA, and AuNPs@PDA-dsDNA measured by DLS.

AuNPs were first synthesized by sodium citrate reduction according to a reported method with some modifications. The TEM image (Fig. 1A) shows that the prepared AuNPs are well-dispersed, and the average diameter is about 13 nm. This data is consistent with the characteristic absorbance peak of AuNPs at 520 nm, as shown in Fig. 1C. The AuNPs were then incubated with dopamine to form AuNPs@PDA with a core–shell structure through the self-polymerization of dopamine under alkaline conditions (pH 8.5). The TEM image (Fig. 1B) reveals that the PDA shell is approximately 4 nm thick and wraps around the surface of the AuNPs; this indicates the successful synthesis of AuNPs@PDA. The DLS data shows that the hydrodynamic diameter of AuNPs increases after coating with PDA (Fig. 1D), which is consistent with the TEM imaging results. Moreover, the NIR absorbance of the prepared AuNPs@PDA is much higher than that of the unmodified AuNPs, which contributes to the absorbance in this range of PDA coating.

PDA has some interesting functional groups such as amino and catechol, which allow it to conjugate with biomolecules containing thiol group through the Michael addition reaction. We then used the prepared AuNPs@PDA to be further functionalized with thiol-terminated DNA, which was pre-hybridized with the FITC-labelled antisense probe. After modification, the maximum absorbance peak of AuNPs@PDA red shifts (Fig. 1C), and the DLS data also shows a further increase in particle size (Fig. 1D). These results indicate the successful modification of AuNPs@PDA with thiol-terminated DNA.

To prove that the thiol-terminated DNA is covalently linked to the surface of AuNPs@PDA, the unlabeled DNA has been used as control. As shown in Fig. S1A (in ESI†), the particle size after modification with thiol-terminated DNA is much larger than the size after modification with unlabeled DNA. It may be because only one end of the thiol-terminated DNA is linked to the surface of AuNPs@PDA, and the remaining part is outside the surface through electrostatic repulsion between each other. On the other hand, most of the unlabeled DNA was attached to the surface of nanoparticles by adsorption. Furthermore, thiol-terminated DNA occupied less space on the surface of AuNPs@PDA and thus more of them could be linked. This statement is consistent with the results shown in Fig. S1B (in ESI†); this further indicates the successful covalent modification of nucleic acid probes on the surface of AuNPs@PDA (AuNPs@PDA-dsDNA). It was estimated by the standard curve (Fig. S2 in ESI†) that approximately 48 thiol-terminated DNA was covalently linked to the surface of each particle.

### The nanoprobe for *in vitro* fluorescence response to the miR-21 target

**Fig. 2:**
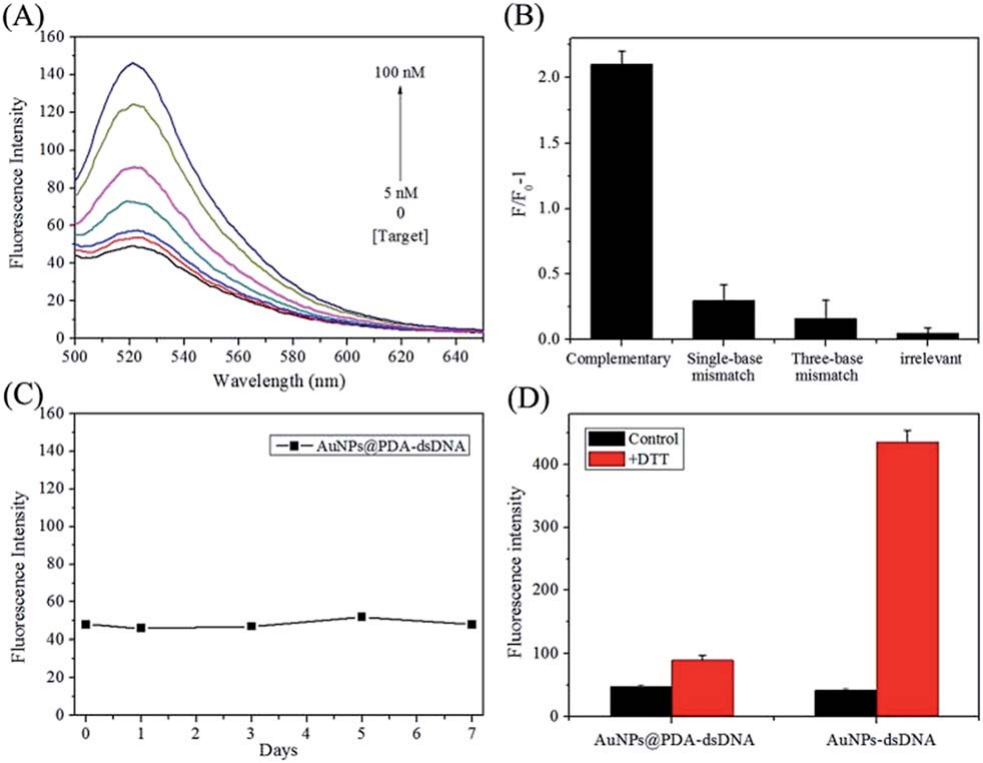
(A) Fluorescence-emission spectra of the AuNPs@PDA-dsDNA solution upon the addition of different concentrations of miR-21 target; (B) fluorescence response (*F*/*F*_0_ − 1) of AuNPs@PDA-dsDNA towards the complementary target, single-base mismatch target, three-base mismatch target, and irrelevant sequence. *F*_0_ and *F* are fluorescence intensities in the absence and the presence of a target, respectively. The data shown represent the average of three independent experiments; (C) the time-dependent fluorescence intensity changes of the AuNPs@PDA-dsDNA solution; (D) the fluorescence intensity changes of AuNPs-dsDNA and AuNPs@PDA-dsDNA solutions after being treated with DTT. The data shown represent the average of three independent experiments.

It has been reported that PDA, AuNPs, and AuNPs@PDA can all serve as efficient quenchers for fluorophores. In this study, AuNPs@PDA was expected to quench the fluorescence of an FITC-labelled antisense probe, which was hybridized to thiol-terminated DNA and FITC was in close proximity to the particle surface. Fig. 2A shows the fluorescence-emission spectra of a AuNPs@PDA-dsDNA solution upon the addition of different concentrations of miR-21 target. We can find that the AuNPs@PDA-dsDNA itself exhibits weak fluorescence emission, which may be due to the fluorescence quenching of FITC caused by AuNPs@PDA. Upon the addition of the miR-21 target, the FITC-labelled antisense probe can hybridize to the target through a branch migration process. Thus, their hybrids can release from the nanoparticle surface, leading to fluorescence restoration. As shown in Fig. 2A, an obvious increase in fluorescence intensity can be found as the concentration of the miR-21 target is increased from 5 nM to 100 nM.

This nanoprobe also exhibited high selectivity for the recognition of the miR-21 target. As shown in Fig. 2B, the AuNPs@PDA-dsDNA solution had little fluorescence response to the non-specific target, containing single-base mismatch target, three-base mismatch target, and irrelevant sequence. However, significant fluorescence enhancement was observed with the addition of the complementary miR-21 target. This result clearly indicates that the prepared AuNPs@PDA-dsDNA shows high selectivity to target miRNA and can even distinguish a single-base mismatch.

It is worth mentioning that the prepared AuNPs@PDA-dsDNA exhibits high stability. The fluorescence intensity of AuNPs@PDA-dsDNA can remain unchanged for a long time; this indicates that almost no FITC-labelled antisense probes are released from the nanoprobes (Fig. 2C). The result shown in Fig. 2D shows that the FITC-labelled DNA can detach from the AuNP surface after the addition of DTT; this results in a dramatic fluorescence enhancement. However, the fluorescence of AuNPs@PDA-dsDNA was just slightly increased after the addition of same concentration of DTT. These results indicate that the prepared AuNPs@PDA-dsDNA exhibits high stability due to PDA-assisted surface modification.

### The nanoprobe for miRNA response-based fluorescence imaging

**Fig. 3:**
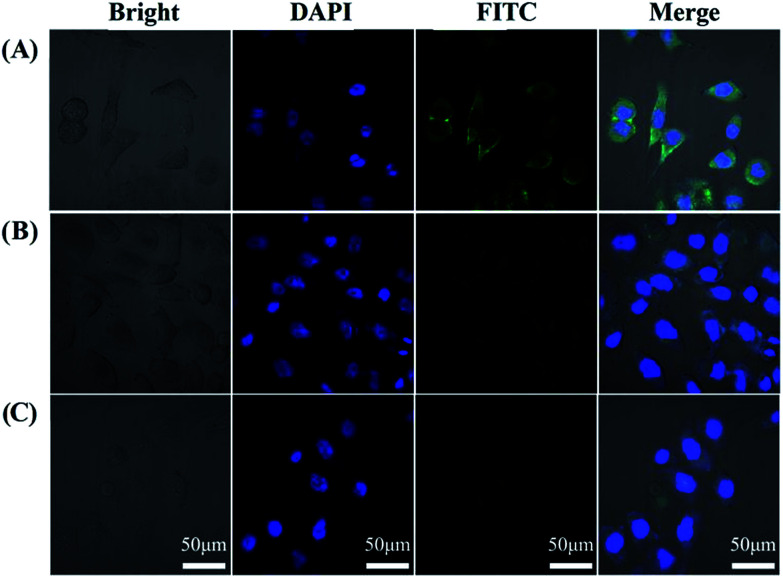
Confocal images of HeLa cells with different treatments: (A) cells incubated with AuNPs@PDA-dsDNA (1 nM) for 2 h; (B) cells incubated with the control nanoprobe containing mismatched antisense oligonucleotide (1 nM) for 2 h; and (C) cells only. The cells were then fixed with 4% paraformaldehyde and stained with DAPI before imaging.

The *in vitro* fluorescence response and the specific recognition ability of AuNPs@PDA-dsDNA provide the possibility for fluorescence imaging of miRNA expressed in living cells. It has been reported that miR-21 is over-expressed in many tumor cells. As shown in Fig. 3A, when incubated with the prepared AuNPs@PDA-dsDNA, the HeLa cells showed bright green fluorescence under excitation at 488 nm. The activation of FITC fluorescence suggests that the prepared AuNPs@PDA-dsDNA can successfully enter HeLa cells. Moreover, the target miRNA expressed in HeLa cells can react with FITC-labelled antisense probes; this results in fluorescence restoration. The clear cytoplasmic localization is consistent with the fact that the mature miRNA is generated in the cytoplasm that is processed by the nuclease Dicer.

To investigate the specificity and stability of the prepared AuNPs@PDA-dsDNA for intracellular microRNA imaging, HeLa cells were incubated with the control nanoprobe containing mismatched antisense oligonucleotide under the same condition as used for AuNPs@PDA-dsDNA. We found that the green fluorescence of these cells was much lower (Fig. 3B); this indicated that little FITC-labelled oligonucleotides were released from the nanoprobe. This result indirectly indicates that the prepared nanoprobe still shows good stability under intracellular conditions. A comparison between the results of Fig. 3A and B demonstrates that the prepared AuNPs@PDA-dsDNA can specifically recognize the target miRNA in cells for fluorescence imaging. We further investigated the fluorescence imaging of the prepared nanoprobes in HepG2 cells. As shown in Fig. S3 (in ESI†), obvious fluorescence enhancement in HepG2 cells was observed. This result is consistent with the fact that miR-21 is overexpressed in HepG2 cells. Thus, the PDA-assisted versatile modification of the nucleic acid nanoprobe can be used for the specific imaging of intracellular miRNA.

### Temperature change induced by 670 nm laser irradiation

**Fig. 4:**
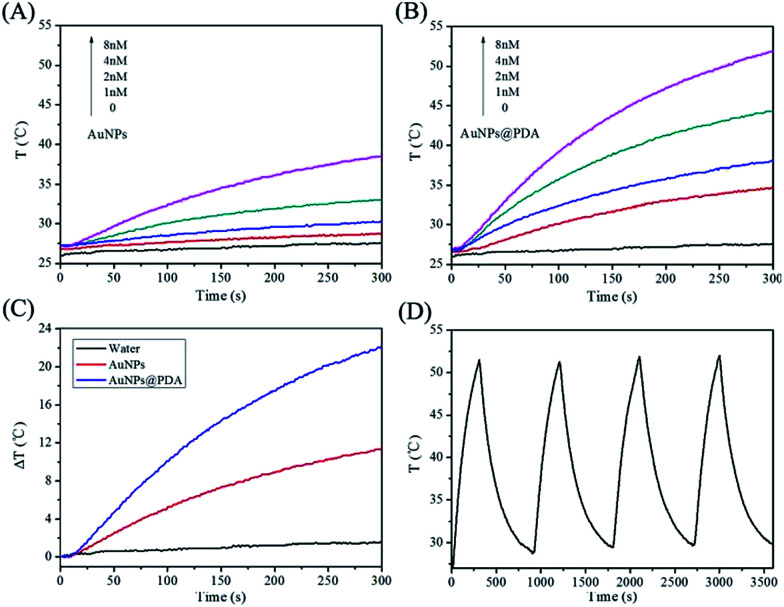
Temperature elevation curves of different concentrations of AuNPs (A) and AuNPs@PDA (B) after exposure to a 670 nm laser (1 W cm^−2^) for 5 min; (C) temperature changes for the same concentration (8 nM) of AuNPs and AuNPs@PDA after exposure to the laser for 5 min; water was used as a control; (D) temperature changes of the AuNPs@PDA solution subjected to four rounds of on/off laser irradiation steps.

Apart from the imaging capability, the high NIR absorbance of the prepared AuNPs@PDA suggests that this nanoprobe may possess high photothermal conversion ability for photothermal therapy. The photothermal conversion effect of AuNPs@PDA was investigated by measuring the temperature change induced by 670 nm laser irradiation. As shown in Fig. 4A, the temperature of the solution with different concentrations of AuNPs@PDA obviously increased after exposure to laser irradiation. The temperature increased up to 52 °C when the AuNPs@PDA concentration increased up to 8 nM, which was sufficient to kill the cancer cells. However, the temperature change of AuNPs with the same concentration was much slower by laser irradiation (Fig. 4B and C). The better photothermal conversion capability of AuNPs@PDA than that of AuNPs may be due to the increased NIR absorbance caused by the PDA coating. These results demonstrate that AuNPs@PDA can be used for photothermal therapy. The photothermal stability of the nanoprobe is also important in photothermal therapy, which we have investigated by subjecting the samples to four on/off laser irradiation steps. The data shown in Fig. 4D indicates that AuNPs@PDA shows good photothermal stability when exposed to repeated laser irradiation.

### Cytotoxicity and photothermal therapy

**Fig. 5:**
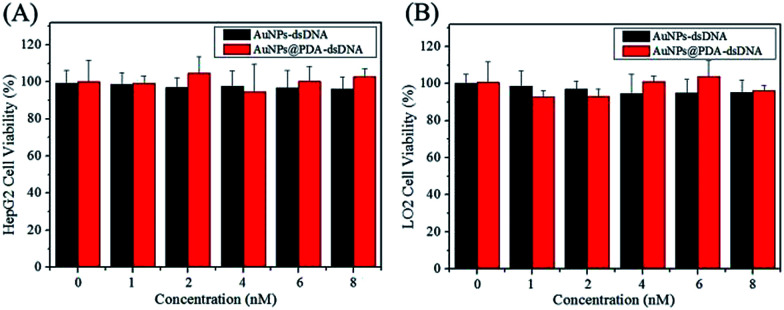
Cell viability of the HepG2 cells (A) and LO2 (B) cells after treatment with different concentrations of AuNPs-dsDNA or AuNPs@PDA-dsDNA for 24 h (without laser irradiation).

**Fig. 6:**
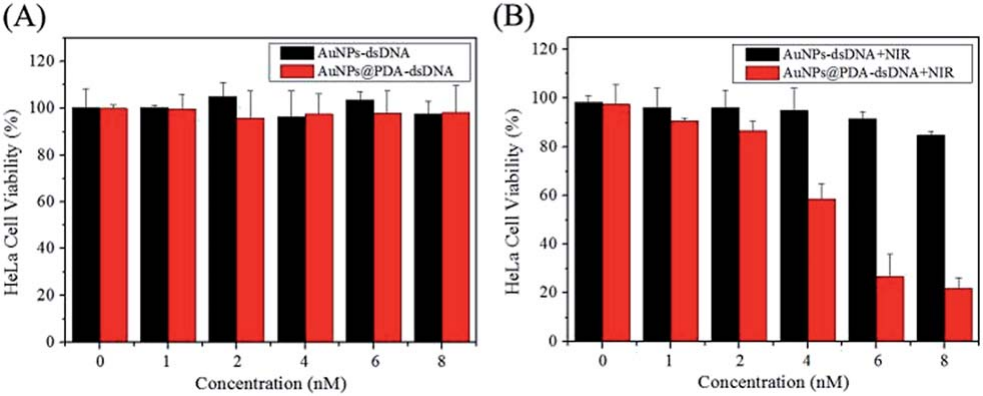
(A) Cell viability of HeLa cells after treatment with different concentrations of AuNP-dsDNA or AuNPs@PDA-dsDNA for 24 h (without laser irradiation); (B) cell viability of HeLa cells after treatment with different concentrations of AuNP-dsDNA or AuNPs@PDA-dsDNA for 2 h and then irradiated with laser for 5 min.

The biocompatibility of a nanoprobe is another important aspect for photothermal therapy. The CCK-8 kit was used to investigate the cytotoxicity of AuNPs-dsDNA and AuNPs@PDA-dsDNA, and the HeLa, HepG2, and LO2 cells were used in this study. As shown in Fig. 5 and 6A, the viability of these cells was still maintained above 90% even after incubation with 8 nM AuNP-dsDNA or AuNPs@PDA-dsDNA for 24 h. These results demonstrated that the prepared nanoprobe exhibited low cytotoxicity to tumor cells and normal cells without laser irradiation.

The photothermal killing ability of AuNPs@PDA-dsDNA was then investigated using CCK-8 and the live–dead viability/cytotoxicity kit and compared with that of AuNP-dsDNA. As shown in Fig. 6A, both AuNP-dsDNA and AuNPs@PDA-dsDNA showed low cytotoxicity to HeLa cells without laser irradiation, and the cell viability both remained above 90%. When exposed to laser irradiation, we found that the AuNPs@PDA-dsDNA exhibited dose-dependent photothermal killing ability. As shown in Fig. 6B, the viability of HeLa cells decreased to about 20% when the concentration of AuNPs@PDA-dsDNA increased up to 8 nM, whereas the HeLa cells incubated with the same concentration of AuNPs@dsDNA still remained highly viable. These results indicate that the modification of PDA can greatly improve photothermal conversion capability and thus enhance the efficacy of photothermal therapy.

**Fig. 7:**
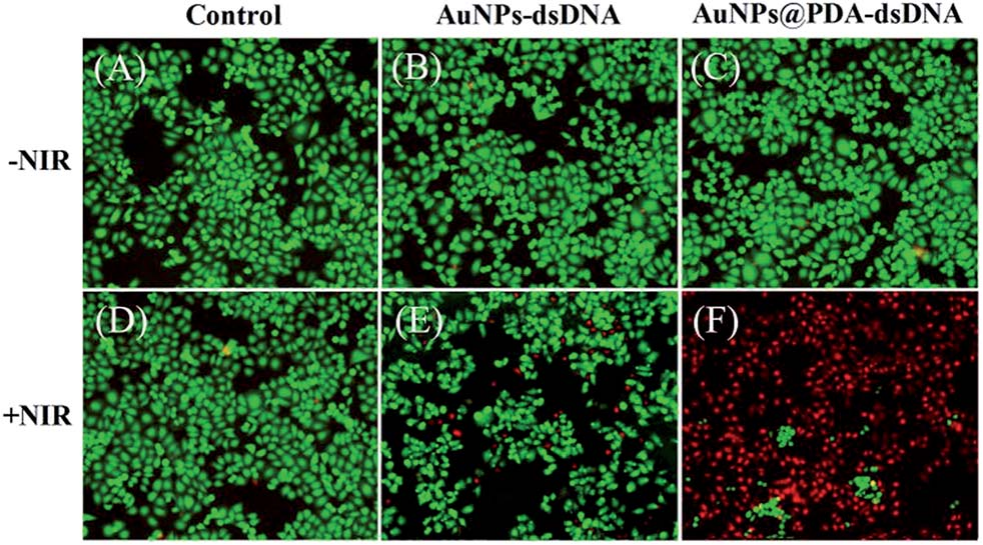
Confocal images of HeLa cells stained with calcein AM (green, live cells) and EthD-1 (red, dead cells). (A) Untreated cells; (B) treated with 8 nM AuNPs-dsDNA; (C) treated with 8 nM AuNPs@PDA-dsDNA; (D) 670 nm laser irradiation for 5 min; (E) treated with 8 nM AuNPs-dsDNA plus laser irradiation; and (F) treated with 8 nM AuNPs@PDA-dsDNA plus laser irradiation.

To further investigate the therapeutic effect of AuNPs@PDA-dsDNA, HeLa cells with different treatments were stained with EthD-1 and calcein AM to image the dead and live cells, which respectively exhibited red and green fluorescence. As shown in Fig. 7, the HeLa cells exhibited almost green fluorescence when exposed to laser irradiation alone or when incubated with the 8 nm nanoprobe alone, thus showing low toxicity of the laser irradiation and the prepared nanoprobe. When simultaneously treated with AuNP-dsDNA and laser irradiation, only a small amount of dead cells were observed with red fluorescence. However, most of the cells exhibited red fluorescence when simultaneously treated with AuNPs@PDA-dsDNA and laser irradiation; this indicated that most of the cells were killed. These phenomena also clearly indicate that the prepared AuNPs@PDA-dsDNA has good therapeutic effect against tumor cells when exposed to laser irradiation.

## Conclusions

In summary, PDA can be used to assist the mild and versatile modification of a nucleic acid probe for miRNA response-based fluorescence imaging and enhanced photothermal therapy. Under slightly alkaline conditions, the PDA-functionalized nanoparticles can be covalently linked with thiol-terminated nucleic acid probes through the Michael addition reaction. This modification is mild and can be performed directly in an aqueous solution, which can better resist hydrolysis than modifications based on active groups such as NHS and maleimide. In this way, the prepared nanoprobe shows good stability and high loading of nucleic acid probes. This type of nucleic acid nanoprobe is also suitable for other nucleic acid response-based imaging and treatment by simply changing the relevant nucleic acid sequence. It is noteworthy that PDA can spontaneously deposit on virtually any surface through self-polymerization; thus, this modification can be combined with many other nanomaterials to prepare nucleic acid nanoprobes with more interesting and attractive functions. It is expected that PDA-based versatile modification can be used to construct a promising platform for tumor imaging and treatment.

## Conflicts of interest

There are no conflicts to declare.

## Supplementary Material

RA-008-C8RA00261D-s001
