# Supplementary material for: Polydopamine-assisted versatile modification of a nucleic acid probe for intracellular microRNA imaging and enhanced photothermal therapy
Source: RSC Adv. 2018 Feb 12;8(13):6781–8. doi: 10.1039/c8ra00261d (PMC9078363; doi:10.1039/c8ra00261d)
Supplement: RA-008-C8RA00261D-s001 [file RA-008-C8RA00261D-s001.pdf]

Electronic Supplementary Material (ESI) for RSC Advances.
This journal is © The Royal Society of Chemistry 2018

Electronic Supplementary Information

Polydopamine assisted versatile modification of nucleic acid probe for intracellular microRNA imaging and enhanced photothermal therapy

Aixian Zheng,[ab] Xiaolong Zhang,[ab] Yanbing Huang,[ab] Zhixiong Cai,[ab] Xiaolong Liu,[ab] and Jingfeng Liu*[abc]

[a.]The United Innovation of Mengchao Hepatobiliary Technology Key Laboratory of Fujian Province, Mengchao Hepatobiliary Hospital of Fujian Medical University, Fuzhou 350025, P. R. China.

[b.]The Liver Center of Fujian Province, Fujian Medical University, Fuzhou 350025, P. R. China.

[c.]Liver Disease Center, The First Affiliated Hospital of Fujian Medical University, Fuzhou 350005, P. R. China.

*Corresponding author. Tel: +86-591-83705927; E-mail: drjingfeng@126.com

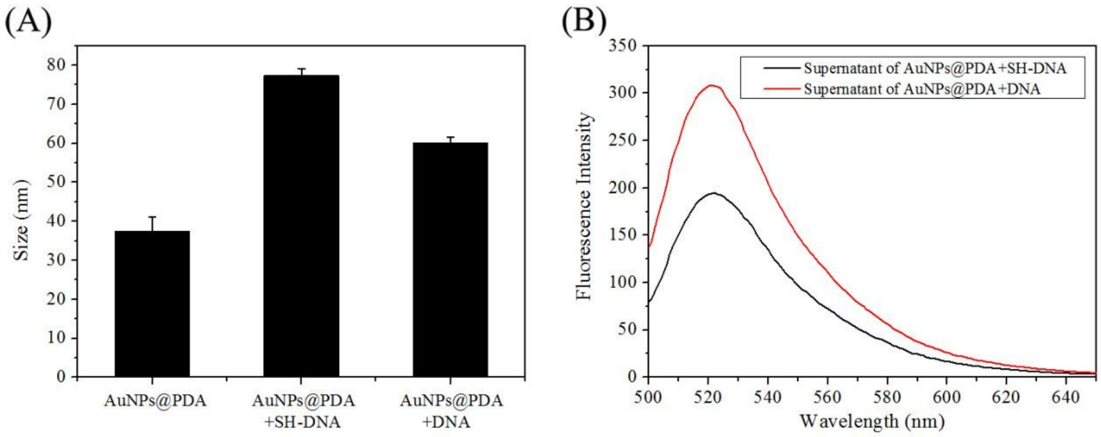

Fig. S1 (A) Hydrodynamic size of AuNPs@PDA and the size after modification with thiol-terminated DNA or unlabeled DNA; (B) The Fluorescence-emission spectra of the diluted supernatant of AuNPs@PDA reacted with thiol-terminated DNA or unlabeled DNA.

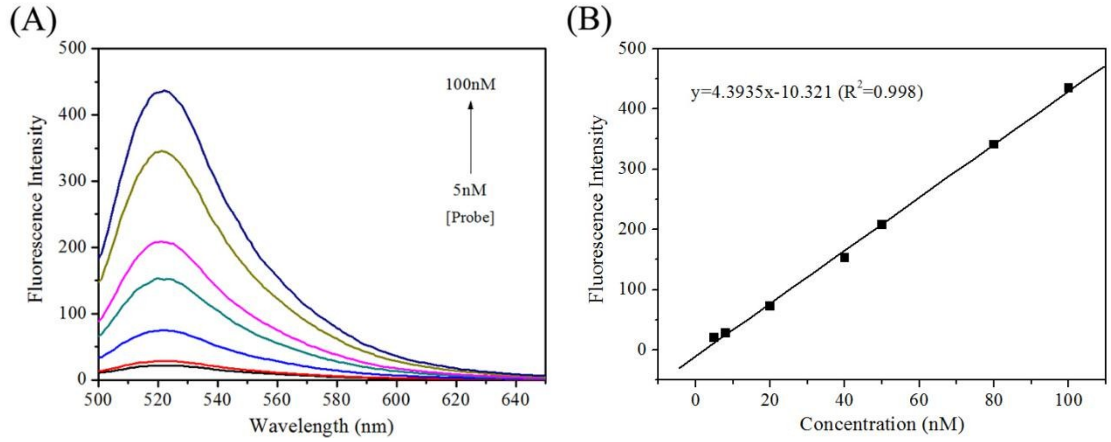

Fig. S2 (A) The Fluorescence-emission spectra of different concentrations of FITC-labelled antisense probe in PBS; (B) the calibration curve for the quantitation of nucleic acid probe.

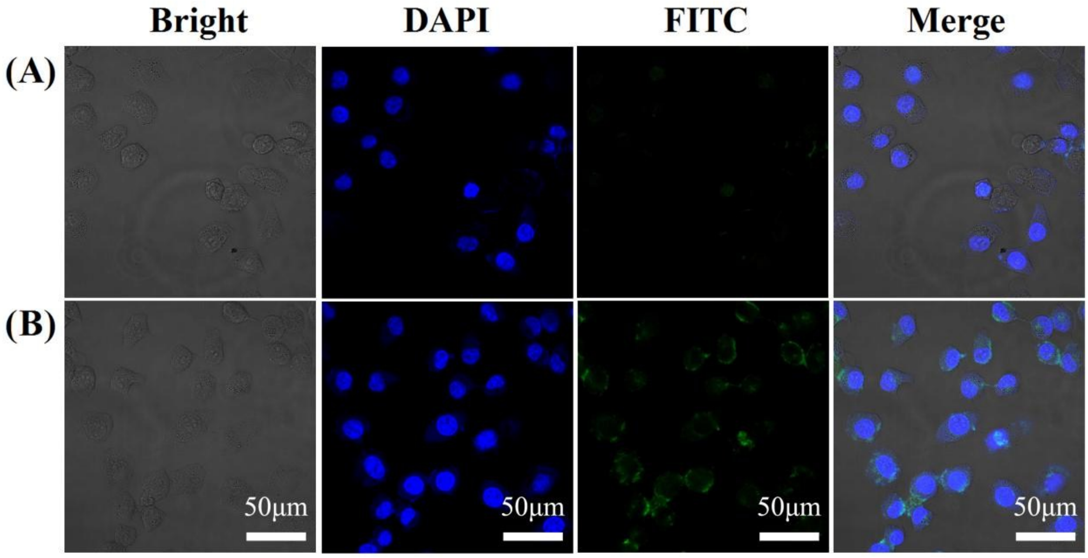

Fig. S3 Confocal images of HepG2 cells with different treatment. (A) cells only; (B) cells incubated with AuNPs@PDA-dsDNA (1 nM) for 2 h. The cells were then fixed with 4 % paraformaldehyde and stained with DAPI before imaging.
